# The hexosamine biosynthesis pathway-related gene signature correlates with immune infiltration and predicts prognosis of patients with osteosarcoma

**DOI:** 10.3389/fimmu.2022.1028263

**Published:** 2022-10-06

**Authors:** Zexin Su, Chenyang Wang, Runsang Pan, Hongbo Li, Junkai Chen, Jianye Tan, Xiaobin Tian, Tiao Lin, Jingnan Shen

**Affiliations:** ^1^ Department of Musculoskeletal Oncology Center, The First Affiliated Hospital of Sun Yat-sen University, Guangzhou, China; ^2^ Guangdong Provincial Key Laboratory of Orthopedics and Traumatology The First Affiliated Hospital of Sun Yat-sen University, Guangzhou, China; ^3^ Department of Neurosurgery, The National Key Clinical Specialty, The Engineering Technology Research Center of Education Ministry of China, Guangdong Provincial Key Laboratory on Brain Function Repair and Regeneration, Zhujiang Hospital, Southern Medical University, Guangzhou, China; ^4^ School of Basic Medicine, Guizhou Medical University, Guiyang, China; ^5^ Department of Orthopedics, The Second Affiliated Hospital of Nanchang University, Nanchang, China; ^6^ Department of Orthopedics, The Affiliated Hospital of Guizhou Medical University, Guiyang, China

**Keywords:** osteosarcoma, metabolism, hexosamine biosynthesis pathway, tumor immune microenvironment, immunotherapy

## Abstract

**Objectives:**

Osteosarcoma is a malignant bone tumor with poor outcomes affecting the adolescents and elderly. In this study, we comprehensively assessed the metabolic characteristics of osteosarcoma patients and constructed a hexosamine biosynthesis pathway (HBP)-based risk score model to predict the prognosis and tumor immune infiltration in patients with osteosarcoma.

**Methods:**

Gene expression matrices of osteosarcoma were downloaded from the Therapeutically Applicable Research to Generate Effective Treatments (TARGET) and Gene Expression Omnibus (GEO) databases. GSVA and univariate Cox regression analysis were performed to screen the metabolic features associated with prognoses. LASSO regression analysis was conducted to construct the metabolism-related risk model. Differentially expressed genes (DEGs) were identified and enrichment analysis was performed based on the risk model. CIBERSORT and ESTIMATE algorithms were executed to evaluate the characteristics of tumor immune infiltration. Comparative analyses for immune checkpoints were performed and the Tumor Immune Dysfunction and Exclusion (TIDE) algorithm was used to predict immunotherapeutic response. Finally, hub genes with good prognostic value were comprehensive analyzed including drug sensitivity screening and immunohistochemistry (IHC) experiments.

**Results:**

Through GSVA and survival analysis, the HBP pathway was identified as the significant prognostic related metabolism feature. Five genes in the HBP pathway including GPI, PGM3, UAP1, OGT and MGEA5 were used to construct the HBP-related risk model. Subsequent DEGs and enrichment analyses showed a strong correlation with immunity. Further, CIBERSORT and ESTIMATE algorithms showed differential immune infiltration characteristics correlated with the HBP-related risk model. TIDE algorithms and immune checkpoint analyses suggested poor immunotherapeutic responses with low expression of immune checkpoints in the high-risk group. Further analysis revealed that the UAP1 gene can predict metastasis. IHC experiments suggested that UAP1 expression correlated significantly with the prognosis and metastasis of osteosarcoma patients. When screening for drug sensitivity, high UAP1 expression was suggestive of great sensitivity to antineoplastic drugs including cobimetinib and selumetinib.

**Conclusion:**

We constructed an HBP-related gene signature containing five key genes (GPI, PGM3, UAP1, OGT, MGEA5) which showed a remarkable prognostic value for predicting prognosis and can guide immunotherapy and targeted therapy for osteosarcoma.

## Introduction

Osteosarcoma (OS), a malignant bone tumor affecting the adolescents and elderly, shows a poor outcome because of high metastatic rates. The prevalence of OS indicates that it is the most common malignant bone tumor with a 4.7 per million incidence rate (1). Additionally, more than 30% of these patients show metastases at initial diagnosis. Although OS with an integrated treatment plan shows a 5-year survival rate of 70%, once the patient develops metastasis or the treatment fails, the overall survival rate drops significantly to 20-30% (2). Tracing back to the source, the primary cause of treatment failure and mortality in patients with OS is the high metastatic potential in combination with the high tumoral heterogeneity. The complicated etiology and the high degree of heterogeneity make the prognostic prediction for OS difficult. Moreover, it is also imperative that novel prognostic models be developed for OS, due to limited treatment strategies.

Recent research has shown that cancer is a metabolic disorder. In 1920s, Otto Warburg discovered that tumor cells are mainly dependent on glycolysis even in the presence of abundant oxygen, or the Warburg effect (3). With the development of biochemical and molecular biological techniques, research on the metabolic characteristics of tumor cells has constantly updated the understanding of the phenomenon and mechanism underlying tumor-related metabolic changes across the different stages of tumorigenesis (4). For examples, androgen receptor-mediated metabolic reprogramming in prostate cancer promotes the metabolic conversion in cancer cells to oxidative phosphorylation, further leading to increased dependence of oxidative phosphorylation and lipogenesis (5). In breast cancer, notably, the inhibition of glycolysis promotes the transformation of the breast cancer stem cells from a quiescent, mesenchymal-like state to an epithelial-like state, referred to as epithelial-mesenchymal transition (6). However, interestingly, glucose not only generates energy through metabolic pathways such as glycolysis but also is metabolized by the hexosamine biosynthesis pathway (HBP), leading to the synthesis of uridine diphosphate N-acetyl-glucosamine (UDP-GlcNAc) and plays an important role in the post-translational modification of proteins (7). Cancer cells upregulate the flux of the HBP pathway and expression of UDP-GlcNAc by increasing the uptake of glucose and glutamine, further promoting signaling pathways related to tumorigenesis through protein N-linked and O-linked glycosylation processes (8). Therefore, targeting the metabolic characteristics of cancer is a promising new strategy for tumor treatment. However, the correlation of metabolism reprogramming with prognosis in OS remains poorly understood.

The tumor immune microenvironment (TIME) comprises tumor cells, immune cells, interstitial cells and extracellular components. TIME reflects the characteristics of immune infiltration in the tumor microenvironment and plays an important role in the occurrence and development of tumors (9). Immune cells in the TIME may have anti- or pro-tumor functions, while these two types of cells play different roles across stages of tumor progression and have been proven to be important in predicting the prognosis of OS patients (10). Moreover, immunotherapy represented by immune checkpoint inhibitors (ICIs) significantly extend the overall survival of patients with advanced cancer (11). Therefore, evaluating the TIME characteristics in the development of OS is helpful to improve the prognosis of the patients through individualized immunotherapy.

In this study, we comprehensively assessed the metabolic characteristics of OS patients according to metabolic pathways and further constructed an HBP-based risk score model to predict the prognosis and tumor immune infiltration. Our findings provide new clues for examining the potential molecular mechanisms underlying the link between metabolic reprogramming and tumor immune infiltration, which may help guide the targeted therapy and immunotherapy for OS.

## Methods

### Collection of gene expression datasets

Clinical data and gene expression matrices of OS were downloaded from the Therapeutically Applicable Research to Generate Effective Treatments (TARGET, https://ocg.cancer.gov/programs/target/projects/osteosarcoma) and Gene Expression Omnibus (GEO, https://www.ncbi.nlm.nih.gov/geo/) databases. A total of 86 OS samples with complete survival data acquired from the TARGET database provided by the National Cancer Institute were defined as the training cohort. The GSE21257 dataset was uploaded by Marieke L Kuijjer (12), comprising 53 samples and the corresponding survival data were defined as the verification cohort following integration.

### Gene set variation analysis (GSVA) and survival curves

GSVA was performed for evaluating the pathway-based prognostic signature in OS (13). As for the definition of metabolic pathways, a total of 114 pathways was screened (14) from The Kyoto Encyclopedia of Genes and Genomes (KEGG). The metabolic pathway score of each OS sample was calculated using the R package “GSVA”. Furthermore, the “survival” R packages was used to calculate the impact of metabolic pathway scores on survival using the coxph() function, and P< 0.05 were considered statistically significant.

### Construction of the prognostic HBP-related gene signature

In this study, 7 genes involved in the HBP pathway were used as candidate prognostic biomarkers for prognosis prediction in OS. Based on R package “glmnet”, these candidate biomarkers were used to obtain an optimal prognostic signature for OS following the LASSO-Cox regression analysis (15). Lastly, we built an optimal prognostic model with 5 genes by selecting the penalty parameter λ, correlated to the minimum 10-fold cross-validation of the model. The HBP-related prognostic risk score formula for each patient was as follows:


$$\text{Risk score}={\text{Σ}}_{1}^{n}\left({\text{coef}}_{i}\times {\text{ expr}}_{i}\right)$$

while coef*_i_* is the coefficient of gene *i*, and expr*_i_* is its relative expression. For the Target-OS dataset, the median value of risk scores in all patients was defined as the cut-off and the patients were then divided into two groups — “high-risk” and “low-risk”. The same cut-off value was used for the GEO21257 dataset. In addition, this risk model was tested for survival prediction ability using the R package “ROCR” by analyzing the 1, 3, and 5-year receiver operating characteristic (ROC) values both in the training and verification cohorts.

### Identification of DEGs and functional enrichment analysis

DEGs were identified using the R package “limma”. Genes with a fold-change greater than 1.5 and P-value less than 0.05 were defined as significant DEGs between the high- and low-risk groups. The R package “ggplot2” was used to visualize these results. Gene ontology (GO) and Reactome pathway enrichment analysis were performed for these DEGs using the Database for Annotation, Visualization, and Integrated Discovery (DAVID, https://david.ncifcrf.gov) tool, and FDR< 0.05 was considered statistically significant.

### Immune landscape analysis and prediction of immunotherapy responses

Using an online analytical platform CIBERSORT (https://cibersortx.stanford.edu/), the compositional proportion of immune infiltrating cells in the OS tissues was calculated based on the characteristic gene set of 22 immune cell subtypes (16). According to the relative abundances of 22 immune infiltrating cells, the differences in immune cell infiltration between the high-risk and low-risk groups were analyzed. The ESTIMATE algorithm was utilized to calculate the immune score, stromal score, ESTIMATE score and tumor purity according to the relative abundances of immune infiltrating cells and stromal cells in OS patients. The expression of six common immune checkpoints (PD-1, CTLA-4, TIM3, LAG3, TIGIT and BLTA) were compared according to clusters and risks (17). Furthermore, to predict the immune benefits of ICI therapy, the T-cell exclusion, dysregulation and TIDE scores were calculated using to the TIDE online algorithm (http://tide.dfci.harvard.edu/). P value < 0.05 was regarded as a statistically significant difference between high- and low-risk groups using independent Student’s t-test.

### Construction of predictive nomogram

Nomogram or the alignment diagram is used to visualize the relationship among different variables in the prediction model by constructing a multi-factor regression model and integrating scores according to the contribution of each influencing factor to the outcome events. In the present study, all independent prognostic factors and clinical data were included to construct the prognostic nomogram for prediction of 3- and 5-year overall survival of patients in the Target-OS dataset. Calibration curves were plotted to evaluate the performance of the nomogram.

### Screening key metastasis-associated genes

To screen the key genes in the risk model, the metastatic status was compared between the high- and low-risk groups using the Chi-square test. The ROC curve for metastatic prediction using each HBP-related gene was visualized both in the training and verification cohorts. Genes with AUCs greater than 0.65 were considered the key genes for the prediction of metastasis in OS patients.

### Immunohistochemical analysis

In the present study, the protein expression of UAP1 in OS tissues was assesed by immunohistochemistry (IHC) staining. A total of 56 OS tissues with corresponding clinical follow-up information were obtained from The First Affiliated Hospital of Sun Yat-sen University. All the patients signed an informed consent form. The slides were incubated with anti-UAP1 following the manufacturer’s instructions. The IHC staining scores for UAP1 were assessed by two independent pathologists. Based on the percentage of positively stained cells, the score was calculated as — 1 for 0-25%; 2 for 26-50%; 3 for 51-75%; and 4 for 75-100%. The score of staining intensity was ranged from 0 to 3. The final IHC staining score for each tissue was obtained by multiplying the scores of positively stained cells and the scores of staining intensity. All patients were divided into two groups base on UAP1 expression to plot the overall survival curve and lung metastasis-free survival curve, using P< 0.05 as the significance threshold.

### Drug sensitivity analysis

The gene expression dataset and the drug sensitivity information for NCI‐60 cancer cell lines were obtained from CellMiner (https://discover.nci.nih.gov/cellminer). After data integration, a Pearson correlation analysis between drug sensitivity and the expression of prognostic HBP-related candidate hub genes was conducted. P-value < 0.05 with correlation ≥ 0.30 was considered statistically significant.

### Western blot analysis

Seven human OS cell lines, U2OS, SAOS2, HOS, 143B, SJSA1, MG63 and G292, were obtained from American Type Culture Collection (ATCC). All cell lines were cultured in Dulbecco’s modified Eagle’s medium (DMEM, Gibco, Grand Island, NY, USA) supplemented with 10% fetal bovine serum (Gibco, Grand Island, NY, USA) at 37°C and 5% CO_2_. The protein samples extracted from different OS cells were resolved by SDS-PAGE and transferred subsequently onto polyvinylidene fluoride membranes, and blocked in 5% skim milk at room temperature for 1 h. The membranes were incubated with the primary antibody against UAP1 (Mouse monoclonal, 67545-1-Ig; Proteintech) at 4°C for 6 h, following which, these were incubated with secondary antibodies at room temperature for 1 h. Finally, the immunoreactive signals on the membranes were visualized using an enhanced chemiluminescence kit.

### Cell CCK-8 assay

Four antitumor drugs including cobimetinib, copanlisib, selumetinib, and tamoxifen were purchased from MedChemExpress (Shanghai, China). For the CCK-8 assay, OS cells in the logarithmic growth phase were plated in 96-well plates and treated with different concentrations of drugs. After 72 h of drug induction, 10 µL CCK-8 solution was added to the cells and incubated for 2.5 h. The optical density (OD) at 490 nm was measured on a microplate reader. The IC50 value was calculated on the GraphPad Prism 9 software by non-linear regression analysis.

## Results

### Overexpression of the HBP pathway is related to poor prognosis in OS

**Figure 1:**
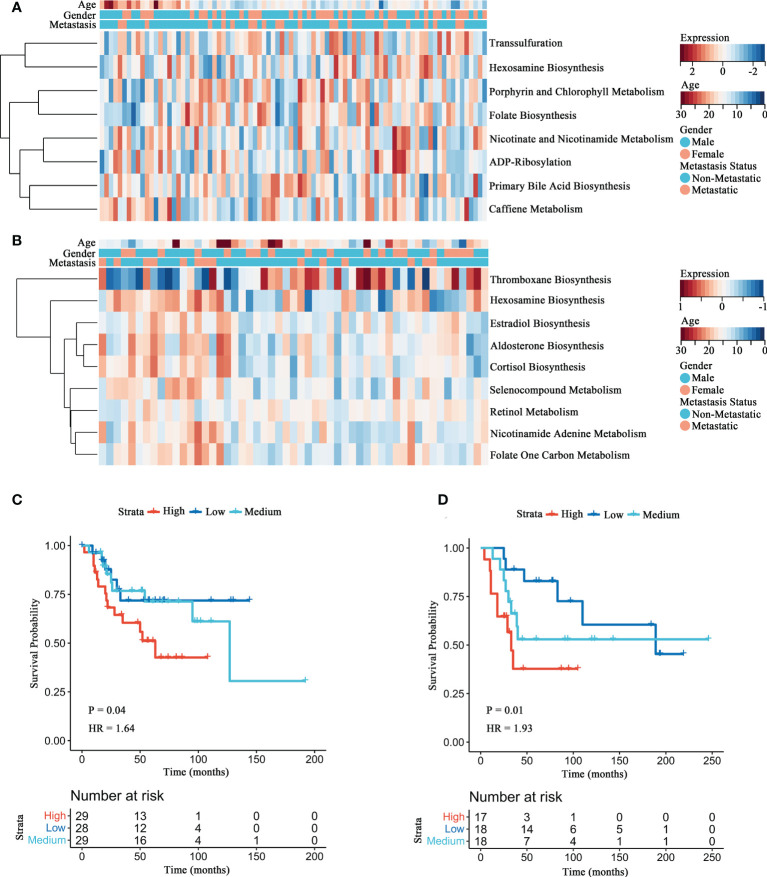
Identification of the HBP pathway associated with overall survival in OS patients. **(A, B)** The heatmaps of survival-associated metabolic signatures in Target-OS **(A)** and GSE21257 **(B)** cohorts. **(C, D)** Survival analyses of the HBP pathway for Target-OS **(C)** and GSE21257 **(D)** cohorts show considerable differences between the high and low GSVA score group.

To detect the distinct metabolism pathways associated with prognosis, GSVA was conducted for 114 metabolic pathways obtained from KEGG. Eight metabolic pathways were screened in the Target-OS (**Figure 1A**), including primary bile acid biosynthesis, caffiene metabolism, transsulfuration, nicotinate and nicotinamide metabolism, hexosamine biosynthesis, porphyrin and chlorophyll metabolism, folate biosynthesis and ADP-ribosylation. In the GSE21257 cohort (**Figure 1B**), nine metabolic pathways were associated significantly with prognosis, including nicotinamide adenine metabolism, aldosterone biosynthesis, cortisol biosynthesis, folate one carbon metabolism, thromboxane biosynthesis, estradiol biosynthesis, retinol metabolism, hexosamine biosynthesis and selenocompound metabolism. The HBP pathway was commonly observed for both datassets and predicted poor prognosis of OS patients. Kaplan-Meier survival analyses of HBP pathway in the Target-OS cohort (**Figure 1C**) and the GSE21257 (**Figure 1D**) cohorts showed that patients with low GSVA scores showed better overall survival than those with high scores (P = 0.044 in Target-OS; P = 0.015 in GSE21257). These results demonstrated that the HBP pathway plays a major role in OS, particularly in the prognosis of OS patients.

### Construction of the HBP-related five-gene signature for prognosis prediction for OS

**Figure 2:**
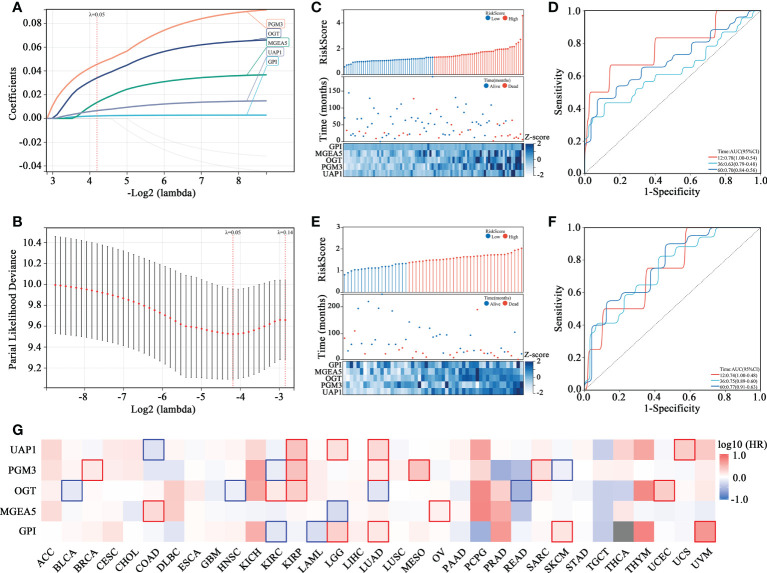
Identification of HBP-related 5 genes with prognostic value in OS patients. **(A, B)** The adaptive LASSO Cox regression for the prognostic value of HBP-related genes. **(C–F)** The risk score distribution, survival status, heatmap and time-dependent ROC curve of HBP-related gene signature in Target-OS **(C, D)** and GSE21257 **(E, F)** cohorts. **(G)** The survival heatmap of HBP-related genes across all tumor types from The Cancer Genome Atlas (TCGA). The red frame and blue frame represent statistical significance in Kaplan-Meier survival analyses.

To assess the prognostic prediction value of the HBP pathway in OS, a risk signature model was constructed (**Figures 2A, B**). Five genes with the best lambda value were selected following LASSO analysis for establishing the risk model, including PGM3, OGT, MGEA5, UAP1 and GPI. A risk score was assigned to each patient according to the constructed prognostic model (Risk score = 0.0026*GPI expression + 0.017*MGEA5 expression + 0.042*OGT expression + 0.057*PGM3 expression + 0.008*UAP1 expression) and patients in the Target-OS (**Figure 2C**) and the GSE21257 (**Figure 2E**) cohorts were categorized into high- and low-risk groups. The levels of gene expression in the HBP-related gene signature and the survival data for each patient in the Target-OS (**Figure 2C**) and GSE21257 (**Figure 2E**) cohorts were visualized on a heatmap. The constructed risk model showed promising predictive ability over a period of 5 years in both cohorts according to ROC analysis, the AUCs for 1-, 3-, and 5 years in the Target-OS cohort was 0.78, 0.63, and 0.70, respectively (**Figure 2D**), while the corresponding values in the GSE21257 cohort were 0.74, 0.75, and 0.77 (**Figure 2F**). Moreover, the survival heatmap of all tumor types from The Cancer Genome Atlas (TCGA) datasets showed significant correlation between HBP-related genes and the overall survival of patients (**Figure 2G**).

### Functional characteristics of DEGs based on the HBP-related risk model

**Figure 3:**
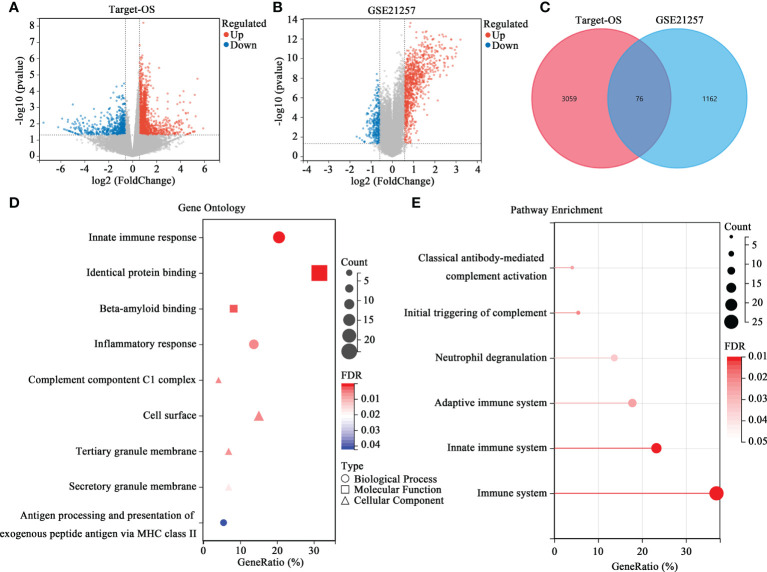
Differential expression analysis and enrichment analysis of the HBP-related prognostic model. **(A, B)** Volcano map of DEGs between the high- and low-risk groups in Target-OS **(A)** and GSE21257 **(B)** cohorts. **(C)** Venn diagram of the intersection DEGs between the Target-OS and GSE21257 cohorts. **(D, E)** Functional annotation of the 76 DEGs in GO function **(D)** and REACTOME pathway enrichment analysis **(E)**.

Further analyses were conducted to reveal how the HBP-related risk model impacted OS patients’ prognoses. First, the levels of gene expression in the high- and low-risk groups were compared to screen DEGs. As shown in **Figure 3A**, 3135 DEGs were identified, among which 2412 were upregulated and 723 were downregulated in the Target-OS cohort. In the GSE21257 cohort, 1238 DEGs were identified, of which 937 were upregulated and 301 were downregulated (**Figure 3B**). The intersection of the Venn diagram shows the number of overlapping genes between both cohorts, and 76 genes were commonly identified (**Figure 3C**). GO function enrichment analysis indicated that DEGs were enriched in innate immune response, identical protein binding, beta-amyloid binding, inflammatory response (**Figure 3D**). The Reactome pathway enrichment analysis indicated that DGEs was closely associated with the immune system, innate immune system, adaptive immune system, neutrophil degranulation, initial triggering of complement and classical antibody-mediated complement activation (**Figure 3E**). Therefore DEGs were strongly associated with immunodeficiency in OS, thereby likely contributing to a poor prognosis in these patients.

### Differential immune cell infiltration correlates with the HBP-related risk model

**Figure 4:**
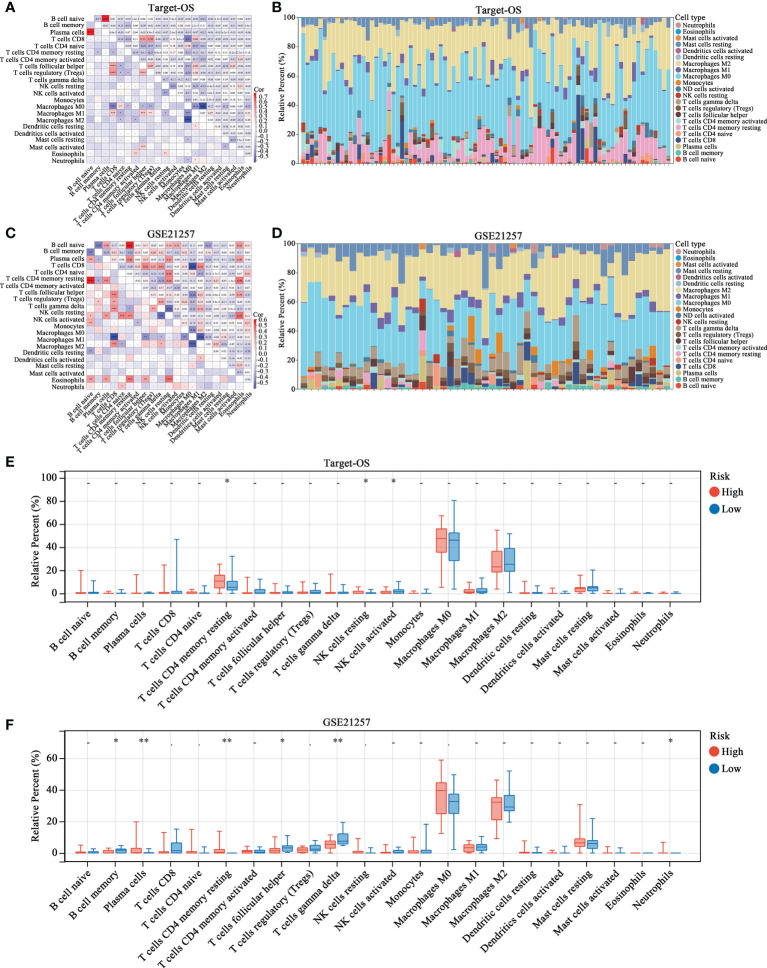
The landscape of immune cell infiltration in high- and low-risk OS patients. **(A–D)** Correlation matrix and relative proportion of all 22 immune infiltration cell proportions based on CIBERSORT in the Target-OS **(A, B)** and GSE21257 **(C, D)** cohorts. **(E, F)** Boxplots visualize the differences of all 22 immune cells between the high- and low-risk groups. “*” represents P < 0.05 and “**” represents P < 0.01.

The proportions of different immune cells types were calculated by CIBERSORT and correlations among immune cells were relatively weak, indicating weak interactions among immune cells in OS (**Figures 4A, C**). After organizing the immune infiltration patterns in patients in the Target-OS (**Figure 4B**) and GSE21257 (**Figure 4D**) cohorts, the high- and low-risk groups showed significantly different immune cell infiltration profiles. The infiltration of resting CD4 memory T cells, resting NK cells and activated NK cells differed significantly between the high- and low-risk group in the Target-OS cohort (**Figure 4E**). Moreover, in GSE21257, the infiltration of plasma cells, resting CD4 memory T cells, follicular helper T cells, gamma delta T cells and Neutrophils differed significantly (**Figure 4F**).

### Immune infiltration characteristics and immunotherapeutic responses correlate with the HBP-related risk model

**Figure 5:**
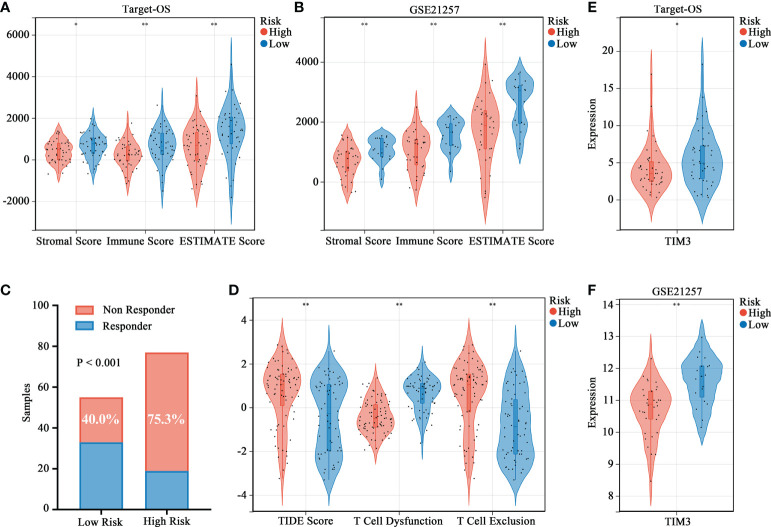
Association between the HBP-related prognostic risk model and immune microenvironment along with immunotherapy prospects. **(A, B)** Stromal, immune and ESTIMATE scores calculated by ESTIMATE algorithm between the high- and low-risk groups. **(C)** Distributions of responders and non-responders predicted by TIDE immunotherapy analyses in high and low risk groups among all patients in Target-OS and GSE21257 cohorts. **(D)** Distribution of TIDE scores in high- versus low-risk group. **(E, F)** Violin plots visualizing significantly different immune checkpoint (TIM3) between high- and low-risk patients. “*” represents P < 0.05 and “**” represents P < 0.01.

The ESTIMATE algorithm was used to evaluate the immune status and patients in the high-risk group showed lower stromal, immune, and ESTIMATE (**Figures 5A, B**) scores. Moreover, the efficacy of immunotherapy evaluated by TIDE analysis showed that patients in the high-risk group were more likely to have no response to immunotherapy as compared to those in the low-risk group (**Figure 5C**). Moreover, the TIDE score and T-cell exclusion score were significantly higher in the high-risk group while the T-cell dysfunction score was low (**Figure 5D**). We assessed the association between risk stratification and several immune checkpoints, including PD-1, CTLA-4, TIM3, LAG3, TIGIT and BTLA. High TIM3 expression was observed in low-risk patients both in Target-OS (**Figure 5E**) and the GSE21257 (**Figure 5F**) cohorts.

### The HBP-related risk model is independent prognostic factor and can predict metastasis in OS

**Figure 6:**
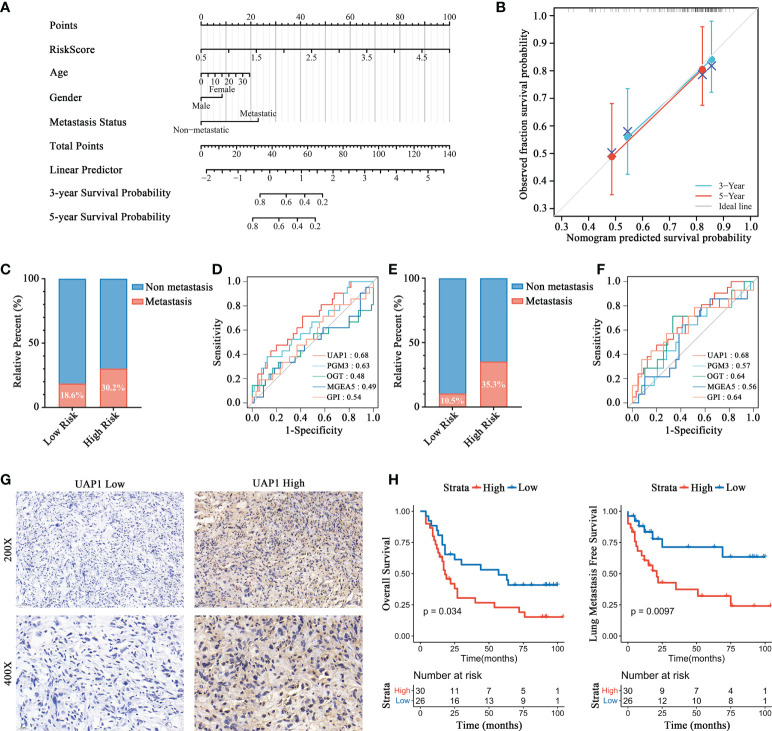
Prognosis prediction validation of HBP-related risk model for OS patients. **(A)** Nomogram for predicting the probability of 3-, and 5-year overall survival for OS patients. **(B)** Calibration plot of the nomogram for predicting the probability of overall survival at 3-, and 5 years. **(C, E)** Distributions of patients with metastasis and non-metastasis between high- and low-risk groups in Target-OS **(C)** and GSE21257 **(E)** cohorts. **(D, F)** ROC curves for evaluating the predictive efficacy of HBP-related genes for metastasis in the Target-OS **(D)** and GSE21257 **(F)** cohorts. **(G, H)** Survival analysis showed a different survival portion between high and low UAP1 expression in osteosarcoma patients for overall survival **(G)** and lung metastasis free survival **(H)**.

By integrating risk score, age, gender, and metastasis status, a quantitative method was developed to create a nomogram model for predicting the overall survival of patients in the Target-OS cohort (**Figure 6A** and **Table 1**). As shown in the calibration plot, a good performance was achieved in predicting 3- and 5-year survival probabilities (**Figure 6B**). In the Target-OS cohort, higher fraction of metastasis was observed (Chi-square = 1.575, P = 0.210) among OS patients in the high-risk group (30.2%) than those in the low-risk group (18.6%) (**Figure 6C**), and consistent results were obtained in the GSE21257 cohort (Chi-square = 3.847, P = 0.049) (**Figure 6E**). On comparing the ROC curves of expressions of HBP-related genes for predicting metastasis, UAP1 expression showed a high AUC score in both cohorts (AUC=0.68, **Figure 6D**; AUC=0.68, **Figure 6F**), indicating that it was better for predicting metastasis relative to the other genes in the HBP-related gene signature. Thus, we further analyzed the protein expression of UAP1 in OS tissues by IHC staining (**Figure 6G**). Patients with high UAP1 expression had worse overall survival and lung metastasis-free survival rates relative to other patients as evidenced by the results of the Kaplan-Meier analysis (**Figure 6H**), indicating that UAP1 was a promising biomarker for predicting prognosis and metastasis in OS patients.

**Table 1:** Univariate and multivariate Cox proportional hazard model for overall survival based on the Target-OS cohort.

Characteristics	Univariate analysis		Multivariate analysis	
	HR (95% CI)	P value	HR (95% CI)	P value
**RiskScore**	4.725 (2.451-9.109)	<0.001	3.475 (1.783-6.773)	<0.001
**Age**	0.988 (0.910-1.072)	0.770	1.032 (0.943-1.129)	0.497
**Gender**				
Male				
Female	1.468 (0.706-3.052)	0.304	1.603 (0.733-3.506)	0.237
**Metastasis Status**				
Non-metastatic				
Metastatic	4.770 (2.285-9.954)	<0.001	3.625 (1.601-8.209)	0.002

### Prediction of drug sensitivity targeting UAP1 and verification on OS cells

**Figure 7:**
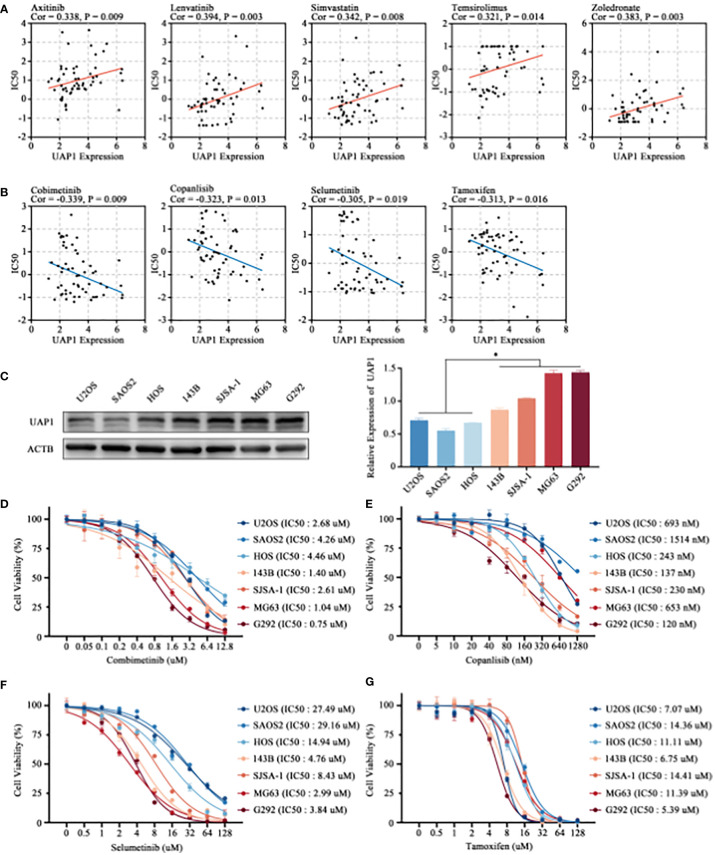
Prediction of drug sensitivity targeting UAP1 and *in-vitro* cell verification. **(A, B)** Correlation analysis between the UAP1 expression and drug sensitivity in the NCI-60 cell lines. **(C)** The level of UAP1 protein expression in OS cell lines, as detected by western blotting. **(D–G)** IC50 of antineoplastic drugs including cobimetinib **(D)**, copanlisib **(E)**, selumetinib **(F)** and tamoxifen **(G)** in OS cell lines according to the CCK-8 assay. “*” represents P < 0.05.

Finally, we analyzed the correlation between UAP1 expression and antitumor drug sensitivity using the CellMiner database. Among the FDA-approved antitumor drugs, UAP1 expression correlated positively with the IC50 of axitinib, lenvatinib, simvastatin, temsirolimus, and zoledronate (**Figures 7A**). Cancer cells with higher UAP1 expression were more sensitive to cobimetinib, copanlisib, selumetinib and tamoxifen (**Figures 7B**). Next, we verified the antineoplastic effect of different concentrations of cobimetinib, copanlisib, selumetinib and tamoxifen on the proliferation of OS cell lines. 7 cell lines were selected and divided into two groups according to the level of UAP1 protein expression (**Figure 7C**), while the low-UAP1 group comprises U2OS, SAOS2 and HOS, and the high-UAP1 group comprises 143B, SJSA-1, MG63 and G292. In the CCK-8 assay, higher IC50s of cobimetinib (P = 0.03) and selumetinib (P = 0.04) were observed in the OS cells with low UAP1 expression (**Figures 7D, F**). Moreover, the IC50s of copanlisib (P = 0.29) and tamoxifen (P = 0.67) among different OS cells showed no statistical differences (**Figures 7E, G**).

## Discussion

In the occurrence of malignancy, tumor cells show some unique malignant characteristics, including uncontrolled proliferation, continuous angiogenesis, tissue invasion and migration. Numerous studies have been conducted on such characteristics and recent studies showed that metabolic reprogramming in tumor cells endows energy and material requirements, thus promoting these malignant features (18). Therefore, researchers have included metabolic reprogramming as a new feature of malignant transformation. To reveal the metabolic reprogramming characteristics in OS, RNA-sequencing data from Target-OS and GSE21257 cohorts were assigned GSVA scores based on 114 metabolic signaling pathways screened from The KEGG database (14). According to the Cox regression analysis, our results indicated that dysregulation in the HBP pathway correlated significantly with the prognosis of OS patients.

The HBP pathway is an important branch of cellular glucose metabolism which impacts the functional macromolecular structures in cancer (19, 20). One downstream metabolite of this pathway, UDP-GlcNAc, is an essential molecule that promote carcinogenesis (21). Here, we constructed an HBP-related prognostic model for OS. The HBP-related prognostic risk score included the expression of GPI, PGM3, UAP1, OGT and MGEA5. GPI catalyzes the production of fructose-6-phosphate through its enzymatic activity in glycolysis. Recently, secreted forms of GPI have been reported frequently in musculoskeletal tumors (22). Anti-apoptotic properties have been observed in cells with high GPI expression owing to resistance to ER stress (23, 24). PGM3 is the enzyme that converts N-acetylglucosamine-6-phosphate to N-acetylglucosamine-1-phosphate. In breast cancer, inhibition of PGMs using the inhibitor FR054 can induce apoptosis by activating the unfolded protein response and promoting the accumulation of intracellular ROS (25). In a study on pancreatic cancer, the upregulation of PGM3 correlated significantly with gemcitabine resistance, while inhibiting PGM3 significantly suppressed the malignant phenotype of tumor cells and enhance the drug sensitivity of gemcitabine by modulating the EGFR-Akt pathway (26). UAP1 is a the rate-limiting enzymes for the production of UDP-GlcNAc, which is an important donor substrate for subsequent glycosylation. UAP1 is overexpressed in prostate cancer cells and correlates negatively with Gleason score. What’s more, UAP1 protects tumor cells from ER stress, thereby conferring advantages for tumor growth (27). UAP1 expression is upregulated in lung adenocarcinoma and correlates positively with larger tumor sizes and advanced TNM stages (28). OGT, a glycosyltransferase, is responsible for catalyzing the O-GlcNAc glycosylation reaction. In breast cancer, reducing intracellular O-GlcNAcation by inhibiting OGT attenuates the expression of the transcription factor FoxM1, further resulting in the downregulation of downstream target genes and inhibiting tumorigenesis (29). Inhibition of OGT also mediate the degradation of HIF-1α by downregulating O-GlcNAcation, further leading to ER stress and inducing apoptosis (30). MGEA5 or OGA is a hexosaminidase responsible for the removal of O-GlcNAc from target proteins. OGA is upregulated in several cancers and drives aerobic glycolysis and tumor growth by inhibiting the catalytic activity of PKM2 (31). However, in another separate study, inhibiting OGA in colorectal cancer cells promoted the level of O-GlcNAcation, thus promoting the expression and activity of β-catenin and E-cadherin and further induce EMT phenotype of cancer cells and promote tumor metastasis (32).

Based on the HBP-related risk model, functional enrichment analysis was performed to investigate the potential underlying molecular mechanisms. DEGs were screened according to risk score grouping and further functional GO and Reactome enrichment analysis were performed. Interestingly, DEGs were significantly enriched in pathways and functions related to immunity. Therefore, we sought to further examine the different immunological characteristics of the high- versus low-risk group. We assessed the proportion of 22 infiltrating immune cells using the CIBERSORT algorithm, which showed the enrichment of M0 macrophages and M2 macrophages in OS patients. Both in the Target-OS and GSE21257 cohorts, a significant increase in the infiltration of resting CD4+ memory T-cells was seen in the high-risk group. Significant difference was obtained by comparative analysis of tumor immune scores including the ESTIMATE, stromal and immune score. The HBP-related prognostic model was closely correlated to tumor immune infiltration in OS patients, which may guide the designing of immunotherapeutic strategies for these patients

Immunotherapy activates or promotes the function of the immune system through targeted drugs and kill cancer cells through the body’s self-defense mechanism. Many solid tumors have been successfully treated with immunotherapy, resulting in a paradigm shift in cancer treatment (33). Therefore, understanding the TIME characteristics can further help develop effective immunotherapy strategies for different cancers. The behavior of immune cells and responses to immunotherapy are intricately linked to various metabolic mechanisms (34). In this study, the HBP-related gene signature could predict the immunotherapeutic effect, as evidenced by the results of the TIDE algorithm, which is commonly utilized for predicting the therapeutic responses of patients undergoing treatment with ICIs (35). The results suggested that patients with higher risk scores had higher TIDE and T-cell dysfunction scores along with lower T cell exclusion score, which implied that higher risk scores were suggestive of less likelihood for the benefits in patients with ICIs owing to immune evasion. Moreover, patients in the low-risk group had higher expression of TIM3, a common inhibitory immunoreceptor identified in cancer during the past decades (36, 37). Overall, these results showed that the HBP-related risk model could guide the immunotherapy for OS patients, and those with lower risk scores may benefit from ICI treatment.

Based on the Cox proportional hazards regression models, univariate and multivariate analyses were performed to identify the prognostic value of the HBP-related risk model for OS patients in the Target-OS cohort. According to the results, our OS risk scoring model shows promising prognostic utility. Moreover, the ROC curves of our HBP-related genes for predicting metastasis and IHC analysis showed that UAP1 expression was a good prognosis predictor not only for overall survival but also for metastasis. Finally, we perform the correlation analysis and experimental verification to investigate the sensitivity of UAP1 to FDA-approved antineoplastic drugs based on the CellMiner database. The results suggested that OS cells with higher UAP1 expression were more sensitive to cobimetinib and selumetinib. In summary, the HBP-related risk model contributes to guide targeted therapy for metabolic reprogramming, and may even help overcome cancer immunotherapy resistance by reversing tumor T-cell exclusion in OS.

## Conclusions

In conclusion, we constructed an HBP-related gene signature containing five key genes (GPI, PGM3, UAP1, OGT and MGEA5) with a remarkable prognostic value for predicting prognosis in OS. Furthermore, significant differences in immune infiltration and immunotherapeutic response were identified between the high- and low-risk patients, which may help guide the development of immunotherapy and targeted therapy for OS.

## Data availability statement

The original contributions presented in the study are included in the article/**Supplementary Material**. Further inquiries can be directed to the corresponding authors.

## Ethics statement

This study was reviewed and approved by the Ethics Committee of The First Affiliated Hospital of Sun Yat-sen University (Approval number: [2021] 755). Written informed consent was obtained from all participants for their participation in this study.

## Author contributions

JS, TL, and XT contributed to the conception and design of the study. ZS, CW, and RP performed the analyses and wrote the manuscript. HL, JC, and JT contributed to parts of the experiments. All authors contributed to the article and approved the submitted version.

## Funding

This work was supported by grants from The National Natural Science Foundation of China (Grant No. 82060491).

## Conflict of interest

The authors declare that the research was conducted in the absence of any commercial or financial relationships that could be construed as a potential conflict of interest.

## Publisher’s note

All claims expressed in this article are solely those of the authors and do not necessarily represent those of their affiliated organizations, or those of the publisher, the editors and the reviewers. Any product that may be evaluated in this article, or claim that may be made by its manufacturer, is not guaranteed or endorsed by the publisher.

## Supplementary material

Supplementary Figure 1 Prognostic value of metabolism pathways in OS patients based on forest plots. **(A, B)** Prognostic value of metabolism pathways in Target-OS **(A)** and GSE21257 **(B)** cohorts.

Supplementary Figure 2 Relationship between the HBP-related risk model and immune checkpoints in OS patients. **(A, B)** Violin plots visualizing expression of the immune checkpoints (BTLA, CTLA4, LAG3, PDCD1, TIGIT) between the high- and low-risk groups in Target-OS **(A)**and GSE21257 **(B)** cohorts.
