# Supplementary figures and images for: The hexosamine biosynthesis pathway-related gene signature correlates with immune infiltration and predicts prognosis of patients with osteosarcoma
Source: Front Immunol. 2022 Oct 6;13:1028263. doi: 10.3389/fimmu.2022.1028263 (PMC9582954; doi:10.3389/fimmu.2022.1028263)

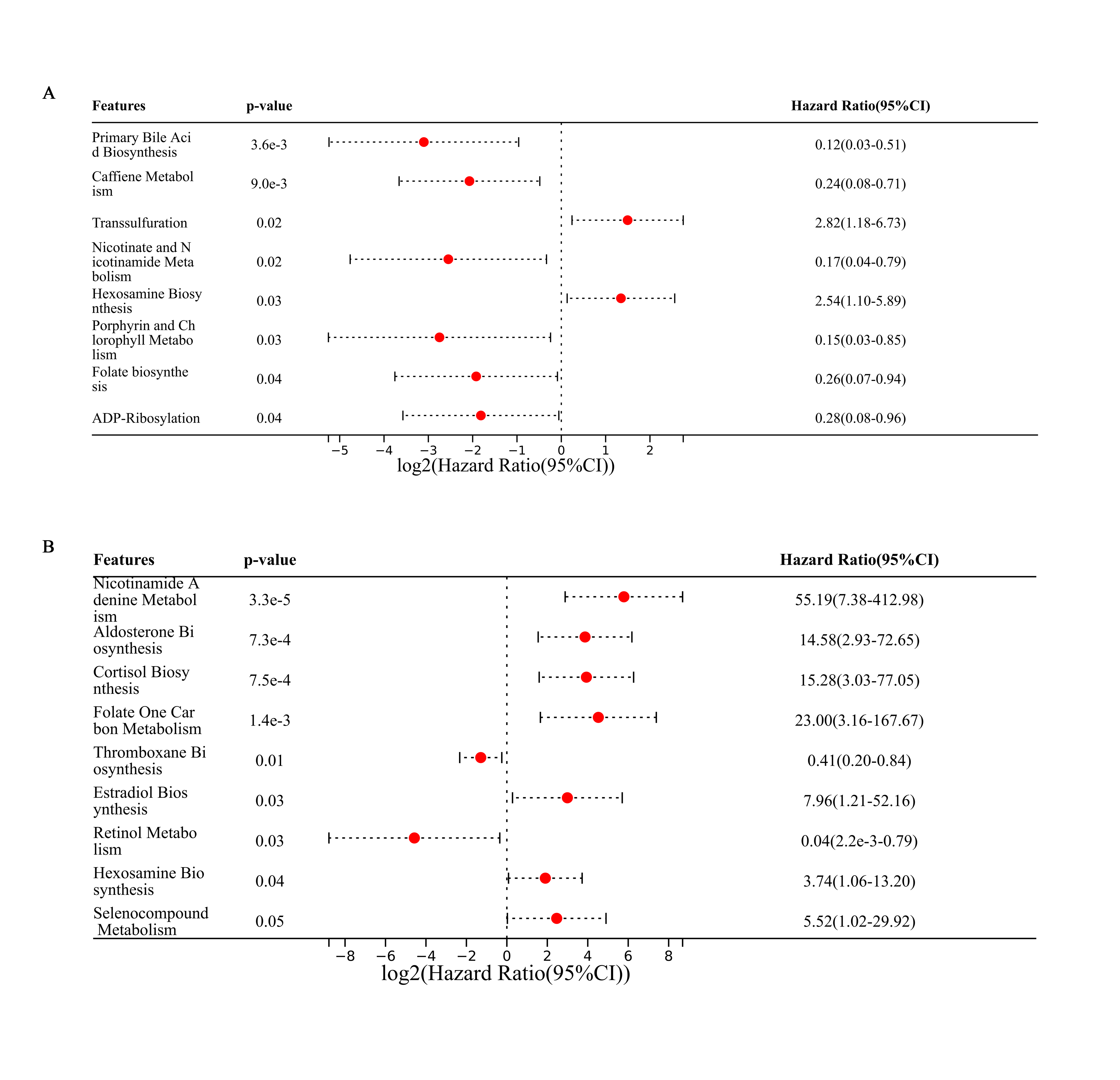

Supplement: Supplementary Figure 1 — Prognostic value of metabolism pathways in OS patients based on forest plots. (A, B) Prognostic value of metabolism pathways in Target-OS (A) and GSE21257 (B) cohorts. [file Image_1.tif]

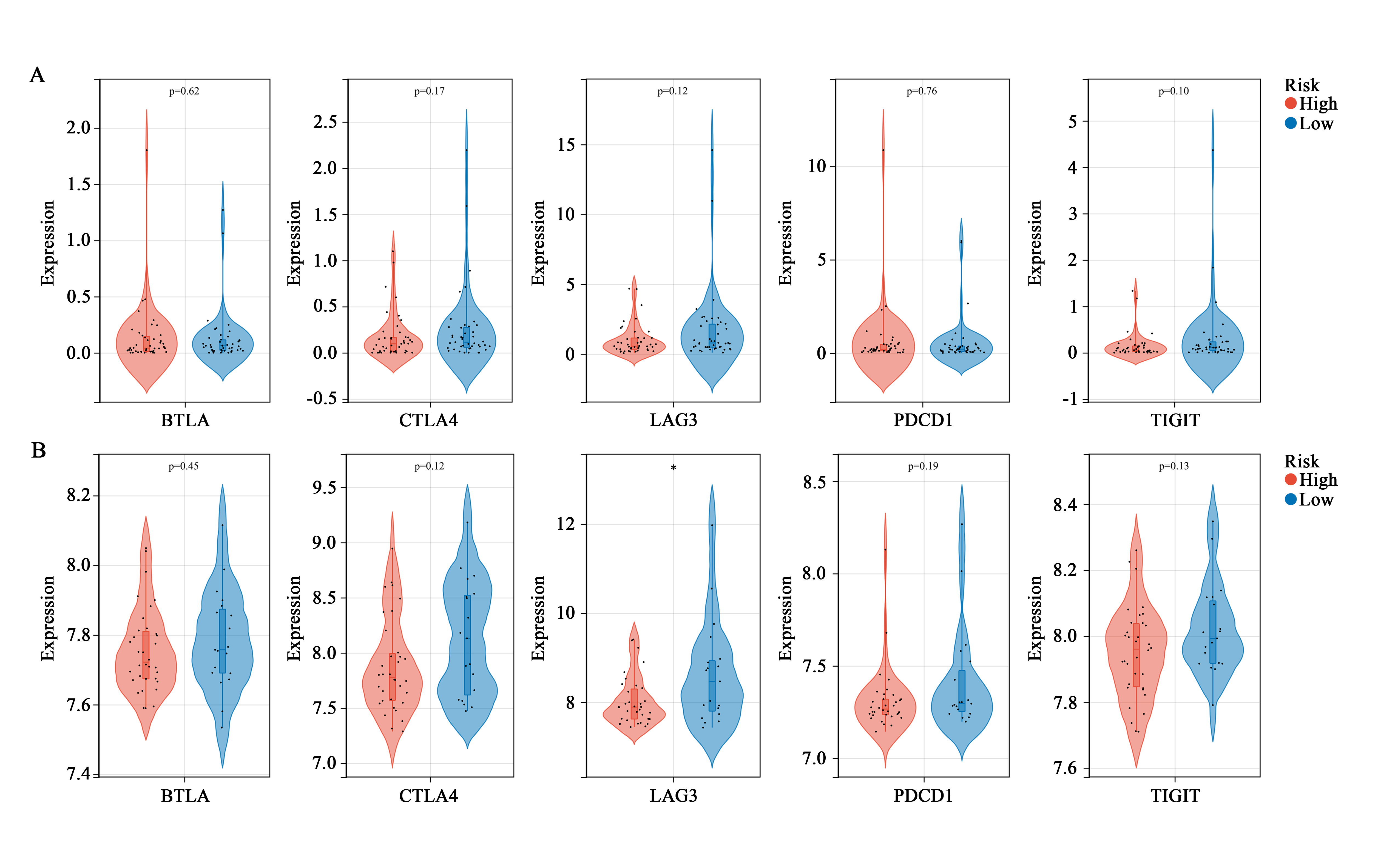

Supplement: Supplementary Figure 2 — Relationship between the HBP-related risk model and immune checkpoints in OS patients. (A, B) Violin plots visualizing expression of the immune checkpoints (BTLA, CTLA4, LAG3, PDCD1, TIGIT) between the high- and low-risk groups in Target-OS (A)and GSE21257 (B) cohorts. [file Image_2.tif]
